# Letter from Mr. Stewart to Dr. Remmett, on the Vaccine Inoculation

**Published:** 1800-03

**Authors:** Thomas Stewart

**Affiliations:** Surgeon. Plymouth


### *Letter from Mr.* STEWART *to Dr.* REMMETT, *on the Vaccine Inoculation. Communicated by Dr.* PEARSON.

DEAR SIR,

I Am happy in having it in my power to communicate *forty-three* fuccefsful cafes of inoculation with vaccine matter; and to thank you for the matter which you fo obligingly fent me. With the lancet you received from Dr. Pearfon, I inoculated the firft cafe; but in no inftance could I fucceed with the thread.*

I fhall do myfelf the honor, at fome future period, to tranfmit two cafes, which go far to prove that cow-pock contagion cannot be communicated by effluvia.

### FIRST CASE.

Mafter Peter Curgenven, a healthy child, aged thirteen weeks, was inoculated with cow-pock matter on Friday, November 1, 1799.

Fourth

---

* The thread was impregnated with matter, and the lancet was ftained at the fame time from the fame puftule.—Dr. PEARSON.

Fourth day, A ſmall elevated ſpot appeared on the part inoculated.

Sixth day, Slight inflammation round the edges of the puſtule.

Seventh day, A beautiful faint bluſh around the inciſed part, of the ſize of a ſeven ſhilling gold coin.

Eighth day, Inflammation increaſing, the puſtule larger than in the inoculated ſmall-pox; ſkin cool.

Ninth day, Slight rigors; the child vomited once; puſtulous fluid limpid, ſkin cool, pulſe natural.

Tenth day, The puſtule beginning to cruſt; no appearance of indiſpoſition.

Eleventh day, Inflammation ſubſiding.

Eighteenth day, The ſcab ſloughed off. There were no eruptions.

Dec. 16th, Maſter Peter Curgenven was inoculated with active variolous matter.

Dec. 17th, Inflammation around the part punctured very conſiderable.

Dec. 18th, Inflammation appears to increaſe.

Dec. 19th, The part inoculated has ſtill an appearance as if the infection had taken.

Dec. 20th, The inflammation ſubſiding.

Dec. 21ſt, Inflammation gone; the child has not been indiſpoſed, nor had any puſtules; his arm is well.

### Second Case.

From the arm of Maſter Peter Curgenven, matter was taken, Nov. 11, and inſerted into both arms of John Luthbridge, of a ſcrophulous habit, aged eighteen months.

Fourth day after inoculation, appearances ſimilar to Maſter Peter Curgenven's.

Fifth day, He became indiſpoſed, and was affected with the common ſymptoms of a patient ſickening with the ſmall-pox.

Sixth day, Several ſmall eruptions on different parts of the body; ſkin cool.

Seventh day, Eruptions increaſed in number and ſize, the puſtules on the inciſed parts alſo increaſing; ſlight inflammation.

Eighth day, The eruptions beginning to diſappear; none of them became purulent; the puſtules and inflammation of the inciſed parts ſtill continue to increaſe; ſlight ſymptoms of fever.

Ninth day, The puſtules on the parts inoculated beginning to cruſt; no complaint.

Twentieth day, The ſcabs ſloughed off.

Dec. 3d, He was inoculated with variolous matter without effect. And, on the 16th, limpid variolous matter was inſerted into both arms, but no diſeaſe enſued.

### Third Case.

On the 15th of November I inoculated Mifs E. Reed with cow-pock matter. The appearance of the arm, &c. fimilar to thofe of Mafter P. Curgenven, until the ninth day, when flight feverifh fymptoms fupervened, and continued until the evening of the tenth day.

Eleventh day, No difeafe.

Dec. 3d, She was inoculated with active variolous matter.

Dec. 9th, She has not taken the infection.

### Fourth Case.

Vaccine matter taken from the arm of Mifs E. Reed, and inferted into the arm of Mary-Ann Moore, aged five years, produced a very mild difeafe.

### Fifth Case.

Mifs Collins, aged eight weeks, was inoculated with matter taken from the arm of Mary-Ann Moore.

Eleventh day, Slight feverifh fymptoms; the puftule on the arm fmaller than in any of the preceding cafes.

Twelfth day, The child appears quite well.

In thirty-eight cafes, the appearances were fo fimilar to the preceding, that I fhould trefpafs on your time to enter farther in detail upon the fubject. There were no eruptions, nor have any fore arms been produced; and the conftitutional affection, or fever, which occurred about the ninth day after inoculation, was much lefs confiderable than even in favourable cafes of inoculated fmall-pox. I am, therefore, firmly of opinion, that the cow-pock inoculation is attended with many advantages fufficient to force its way into general practice here, notwithftanding the oppofition and crafty infinuations of interefted Practitioners.

To obviate the evil intentions of fuch men, the following hiftory is fubjoined:

John M'Cubbin's child, aged two days, on account of his mother being ill with the fmall-pox when he was born, was inoculated with vaccine matter on Tuefday, 21ft of January.

Saturday, 4th day, As it was doubtful whether or not the infection had taken place, it was determined to inoculate him a fecond time with recent matter, which could not, in the courfe of this day, be conveniently procured.

Sunday, 5th day, The punctured part of the right arm is flightly inflamed, and of a puftular form.

Monday, 6th day, The child was very ill all night, with frequent vomiting and bleeding at the nofe, and other fymptoms denoting denoting much conſtitutional diſeaſe. From the bleeding at the noſe, alteration of the countenance, &c. we ſuſpected the ſeat of the diſeaſe to be in the head; there was not, however, the ſlighteſt mark of injury to be perceived externally. He died about one o'clock this afternoon. Upon inſpecting his head, in preſence of Dr. Huggan, we found that by preſſing on the fontanelle and back part of the os frontis, blood iſſued from the noſe.

The ſcalp covering the left parietal bone, and the correſponding part of the occipital, was thickened and diſcoloured, the ſubjacent part of the pericranium to a greater extent was much inflamed. The occipital and left parietal bones, which it covered, were of a dark red colour. Between the dura mater and ſkull of the ſame ſide, there was a great quantity of extravaſated blood; and the brain itſelf underneath was alſo ſlightly inflamed.

That the morbid appearances above deſcribed, were the conſequence of external violence, though that could not be aſcertained, will not be doubted. Nor is it material, whether we ſuppoſe the inflammation or the extravaſation of blood, to have been the immediate cauſe of his death; as this caſe is not communicated with any practical view, but merely, that the unbiaſſed and intelligent part of the profeſſion may judge how much, or rather how little a ſhare the cow-pock had in occaſioning it. As a report has been ſpread abroad, evidently for the purpoſe of diſcouraging the progreſs of the cow-pock, that the child died of this diſeaſe, it was judged neceſſary to deſcribe the morbid appearances, which we diſcovered on diſſection.

I am,

Dear Sir,

With reſpect and eſteem,

Your very humble ſervant,

*Plymouth, Jan.* 31, 1800. THOMAS STEWART, Surgeon.

To Dr. Remmett.

---

### To DOCTOR PEARSON.

My dear Sir,

YOU will be pleaſed with the report which accompanies this letter; and when you have peruſed it, you will have the goodneſs to tranſmit it for inſertion in the next Number of the Medical and Phyſical Journal. For the authenticity of ſeveral of the firſt caſes, I am myſelf reſponſible; and with reſpect to all, I aſſure you, that I know no man better entitled to credit than my reſpectable friend Stewart. I wiſh to add concerning Luthbridge,

Luthbridge, that I never thought the eruption, which took place prior to the attack of the vaccine difeafe, of a variolous nature; and that the boy has now again a fimilar eruption attended with fome degree of fever.

It feems neceffary to publifh the cafe of M<sup>c</sup> Cubbins; and I am glad to inform you, that the mother is recovering from her highly confluent fmall-pox, unfortunate as was the period of attack. Had the child lived, the efficacy of the vaccine infection, in preventing that of the fmall-pox, would, I have no doubt, been ftrikingly manifefted. The cafe would, indeed, have been a very important one. The threads, though ufed by many, have not produced the difeafe in any one inftance within my knowledge. I am,

My dear Sir,

Moft truly, your's,

R. B. REMMETT.

